# Attitudes, hesitancy, concerns, and inconsistencies regarding vaccines reported by parents of preschool children

**DOI:** 10.1590/1984-0462/2023/41/2022009

**Published:** 2023-03-13

**Authors:** Jaime Olbrich, Sandra Regina Leite Rosa Olbrich

**Affiliations:** a Universidade Estadual Paulista “Júlio de Mesquita Filho”, Botucatu, SP, Brazil.

**Keywords:** Vaccines, Attitudes, Parents, Vaccine hesitancy, Vacinas, Atitudes, Pais, Hesitação vacinal

## Abstract

**Objective:**

This study aimed to assess attitudes, concerns, information and knowledge about vaccines among parents of preschool children attending kindergartens in a city in the interior of São Paulo, Brazil, using a self-administered questionnaire.

**Methods:**

Cross-sectional, questionnaire-based study of knowledge and attitudes regarding vaccination among parents of children aged up to 72 months from public and private schools, between 2018 and 2019.

**Results:**

Among the 2,528 questionnaires, 1,261 were answered and grouped by respondents’ educational level. According to information, 96.6% of the children were up to date with vaccines. The prevalence of vaccine hesitancy was 5.0%. The lower the educational level, the lower was the income, the larger the number of household members, and the greater the lack of knowledge about vaccines. The higher the educational level, the lower was the vaccine hesitancy, and the greater the dissatisfaction with the information received.

**Conclusions:**

Generally, parents consider vaccines to be important for preventing diseases and to be safe, with their benefits outweighing the risks. Positive comments were accompanied by doubts, concerns, hesitancy, and inconsistencies. The level of educational attainment makes a difference in the access to information, medical care provided by pediatricians, and the feeling of obligation to vaccinate. Parents have vaccinated and still intend to vaccinate their children, but ensuring adequate levels of vaccination coverage will be a post-pandemic challenge.

## RESUMO

**Objetivo:**

Este estudo procurou avaliar atitudes, preocupações, informações e conhecimentos sobre vacinas por parte de responsáveis por crianças em idade pré-escolar que frequentam instituições de ensino infantil em um município do interior de São Paulo, Brasil, por meio de questionário autoaplicável.

**Métodos:**

Estudo transversal com o uso de questionário dirigido aos pais de crianças de até 72 meses, em escolas públicas e privadas, sobre conhecimentos e atitudes quanto à vacinação das crianças entre 2018 e 2019.

**Resultados:**

Entre 2.528 questionários entregues, 1.261 foram respondidos e agrupados conforme a escolaridade dos entrevistados. De acordo com as informações, 96,6% das crianças estavam com vacinas em dia. A prevalência de hesitação vacinal foi de 5,0%. Quanto menor a escolaridade, menor a renda, maior o número de habitantes na casa e maior o desconhecimento sobre vacinas. Quanto maior a escolaridade, menor hesitação e maior insatisfação com as informações recebidas.

**Conclusões:**

Em geral, os pais consideram as vacinas importantes para prevenir doenças, como proteção, e dizem que os benefícios superam os riscos. Manifestações positivas foram acompanhadas por dúvidas, preocupações, hesitações e inconsistências. O nível de escolaridade faz diferença no acesso a informações, no atendimento médico por pediatras e no sentimento de obrigatoriedade em vacinar. Os pais têm vacinado e ainda pretendem vacinar, mas garantir níveis de cobertura vacinal adequados será um desafio pós-pandemia.

## INTRODUCTION

Vaccines are important to protect the health of children, and mothers usually guarantee compliance with the immunization schedule.^1^ Doubts about safety, number of doses, and vaccine quality, among other factors, can delay acceptance or lead to refusal despite the availability of services — which defines vaccine hesitancy.^2^ The determinants of this behavior are complex, varying across geographic regions and types of vaccine, and are influenced by factors such as confidence, complacency, and convenience, as defined by the Working Group on Vaccine Hesitancy of the World Health Organization (WHO). As a consequence, vaccination coverage rates may decline even in countries with a legal obligation to vaccinate and programs that provide vaccines free of charge like Brazil. The overall vaccination coverage in Brazil was 71.9% in 2018, varying across states from 57.4 to 85.4%.^3^ Brown et al. observed that the main reason for hesitancy in Brazil was the lack of confidence in the vaccines.^4^ Obeying vaccination norms with doubts and reluctance would be a type of hesitancy, and the lack of communication with adequate information between health professionals and parents seems to contribute to this hesitancy.^2,5^ Monitoring parental attitudes, knowledge, doubts and beliefs about vaccines, as well as how and where information is obtained, are important to identify and understand acceptance or hesitancy and to formulate personalized policies.^6^

This study aimed to assess attitudes, concerns, information and knowledge about vaccines among parents of preschool children attending kindergartens in Botucatu, a city in the interior of São Paulo, Brazil, using a self-administered questionnaire.

## METHOD

This was a cross-sectional study conducted from January 2018 to December 2019 in a medium-sized city in the interior of São Paulo, Brazil, using self-administered questionnaires. The questionnaires were handed out to the legal representatives of children aged up to 72 months who attended public or private kindergartens, as proposed in the objective of the study. Among the total number of children, it was estimated a participation of 40%, as a sample size calculation performed for the distribution of questionnaires according to the proportion of municipal public and private schools that were randomly selected. For public schools the city was divided in four sectors — North, South, East and West — and, in each one, the same number of private schools were selected, mainly located in downtown, with almost the same number of children. Larger and smaller schools, in separated groups, were randomly selected.

In each randomly selected school, the study was explained through printed pages, and the individual questionnaires were handed out in sealed envelopes. Inclusion criteria were child’s age up to 6 years (72 months) on the date of the interview, and the respondent signing the Informed Consent Form. Participants who answered fewer than five questions were excluded. The individual questionnaire consisted of 27 questions with multiple choices and some open questions with providing space for free responses. There were 12 questions regarding general information about the child and his/her immunization, including adverse events and authorization to photograph the vaccine card — the objective was to compare if, among respondents, the percentage who answered the cards were up to date was significantly higher than among those who did not authorize). Additionally, there were 15 questions to assess attitudes, hesitancy, vaccination concerns, and sociodemographic information. The questionnaire also contained a space for questions from the participants. When asked about certainty regarding vaccinating the child, the following answers were defined as hesitancy: “I was not sure and so I did not vaccinate”, “I was sure and did not vaccinate”, or “I was not sure and I postponed some vaccines”.

Data were analyzed considering the level of educational attainment of the respondents since we considered this to be the best parameter related to choices and decisions about vaccination. The educational level was classified as: incomplete elementary school (IES), complete elementary school (CES), incomplete high school (IHS), complete high school (CHS), incomplete higher education (IHE), and complete higher education (CHE). The answers were compared across the educational levels to evaluate attitudes and consistency.

For statistical analysis, proportions were compared using Fisher’s exact test and the Cochran-Mantel-Haenszel test, considering p<0.05 to be significant.

The governing bodies responsible for the kindergartens and the local Research Ethics Committee approved the study (CAEE 91408318.1.0000.5411). The Strengthening the Reporting of Observational Studies in Epidemiology statement was followed whenever possible.^7,8^

## RESULTS

A total of 2,528 questionnaires were handed out, 78.1% at public kindergartens and 21.9% at private ones. Of these, 1,939 were returned and 1,261 (65%) met the inclusion criteria, with no difference between public or private schools. A total of 1,208 respondents reported their educational level: 75 (6.21%) had IES, 94 (7.78%) had CES, 168 (13.91%) had IHS, 472 (39.07%) had CHS, 99 (8.2%) had IHE, and 300 (24.83%) had CHE. Of the 1,261 respondents, 53 (4.2%). The percentage of parents answering the questionnaires was lower in the groups with lower educational attainment (p<0.05). Of this sample, 85% of the children were enrolled in public kindergartens. Parental educational level was significantly higher in private schools (p<0.05).

The mean age of the children was 41.7 months (range 3–72). Mothers were the predominant respondents to the questionnaires (90.9%), regardless of educational level. Respondents in the CHE group were significantly older, with a mean age of 35.1 years compared to all the other groups (p<0.001). A higher income, a smaller number of household members, and children with routine follow-up visits to pediatrician were more frequent in the CHE group (p<0.001).

The percentage of respondents who were not satisfied with the information received at the time of vaccine application was higher in the CHE group compared to the other groups (p<0.001). Vaccine reactions were reported by 450 respondents (36.3%), with the pentavalent vaccine (Diphtheria, Tetanus, Pertussis, Hepatitis B, Haemophilus Influenzae Type B) being the most frequently cited (18.1%).

Among respondents who authorized taking a photograph of the vaccination card, 96.6% reported that their children’s vaccines were up to date, and the percentage was the same among those who did not authorize but claimed to be up to date. Some respondents (n=168) attached a copy of the child’s vaccination card, although it was not requested, and all were up to date. In Table 1, the answers are distributed according to the parents’ educational level, and address general information about vaccines and health services. Table 2 presents the considerations about individual and collective protection.

**Table 1.** Vaccine status, services used and decision to vaccinate or not reported by the respondents according to their educational level.

Question	Responses (%)	Educational level*						Overall
		IES	CES	IHS	CHS	IHE	CHE	
Are your child’s vaccines up to date?	Yes	94.7^a^	96.8^a^	96.4^a^	96.8^a^	98.0^a^	96.0^a^	96.5^a^
	No	5.3	3.2	3.0	3.2	2.0	4.0	3.4
	Did not get vaccinated	0	0	0.6	0	0	0	0.1
Where does your child get vaccinated (service)?	Only SUS	100.0^a^	98.9^a^	100.0^a^	97.9^a^	91.9^b^	73.2^c^	90.8
	Only private	0	0.0	0	0.4	1.0	7.0	2.1
	SUS and private	0	1.1	0	1.7	7.1	19.7	7.1
How many vaccines did your child get?	Reported	36.0	28.7	45.2	57.2	54.6	59.7	52.4
	Does not know	64.0^a^	71.3^a^	54.8^ab^	42.8^bc^	45.5^c^	40.3^d^	47.6
How many diseases is your child protected against?	Reported	21.3	20.2	31.0	37.9	44.4	58.7	40.2
	Does not know	78.7	79.8	69.1	62.1	55.6	41.3	59.8
Has your child ever had a reaction to vaccines?	Yes	24.0^a^	26.6^a^	30.5^a^	32.2^a^	43.4^b^	49.7^b^	36.3
	No	76.0	73.4	69.5	67.8	56.6	50.3	63.7
Who decides about the vaccination?	Mother and father	17.3	31.2	29.8	38.2	36.4	56.9	39.7
	Predominantly the mother	82.7^a^	68.8^ab^	70.2^ab^	61.8^b^	63.6	43.1^c^	60.4
Can we take a picture of the vaccine card?	Yes	0	0	0.6	0	0	0	0.1
	No	100.0^a^	98.9^a^	100.0^a^	97.9^a^	91.9^b^	73.2^c^	90.8

IES: incomplete elementary school; CES: complete elementary school; HIS: incomplete high school; CHS: complete high school; IHE: incomplete higher education; CHE: complete higher education; SUS: Unified Health System, the Brazilian public health system. *Percentages followed by the same letter (a, b, c, d) do not differ at the 5% level.

**Table 2.** Knowledge about the importance of vaccination for the individual and collective prevention of diseases according to the educational level of parents with children up to 72 months of age.

Question	Responses (%)	Educational level*						Overall
		IES	CES	IHS	CHS	IHE	CHE	
Would you say: my child may have a severe illness if he or she is not vaccinated?	I strongly agree	63.0	76.3	65.1	70.8	72.5	83.7	73.3
	I partially agree	13.7	15.1	13.9	17.2	23.5	14.2	16.1
	I do not agree	2.7	2.2	4.2	2.8	1.0	1.4	2.4
	I don’t know	20.6^a^	6.5^b^	16.9^a^	9.3^b^	3.1^c^	0.7^c^	8.2
Would you say: it is important to vaccinate my child to prevent the spread of the disease in the community?	I strongly agree	70.3	76.3	72.9	75.4	80.6	87.1	78.1
	I partially agree	13.5	19.4	16.9	16.7	17.4	10.2	15.2
	I do not agree	0.0	1.1	0.6	2.8	1.0	2.0	1.8
	I don’t know	16.2^a^	3.2^b^	9.6^ab^	5.1^bc^	1.0^cd^	0.7^d^	4.9
If children are not vaccinated, what is the risk that they will have a disease that vaccines prevent?	Very high	72.2^a^	71.3^a^	72.5^a^	60.3^ab^	59.6^ab^	55.3^b^	62.3
	Low	2.8	2.1	2.4	4.1	4.0	5.5	3.9
	High	20.8^a^	25.5^a^	22.2^a^	33.7^b^	33.3^ab^	37.5^b^	31.6
	Very low	4.2	1.1	3.0	1.9	3.0	1.7	2.2
How important do you think immunizations are in keeping children healthy?	Extremely important	78.4^a^	83.7^a^	80.2^a^	78.2^a^	84.7^a^	77.7^a^	79.3
	Important	0.0	1.1	0.6	0.2	0.0	0.0	0.3
	Fairly important	0.0	2.2	0.0	0.6	2.0	1.0	0.8
	Not important	21.6	13.0	19.2	21.0	13.3	21.3	19.6
If only your child is not vaccinated, what is the risk of having a disease that vaccines prevent?	Very high	63.5^ab^	68.1^a^	64.1^a^	54.9^bc^	44.4^cd^	37.6^d^	52.6
	Low	5.4	8.5	5.4	7.7	15.2	21.0	11.2
	High	31.1	22.3	29.3	35.1	37.4	37.0	33.7
	Very low	0.0	1.1	1.2	2.3	3.0	4.4	2.5

IES: incomplete elementary school; CES: complete elementary school; HIS: incomplete high school; CHS: complete high school; IHE: incomplete higher education; CHE: complete higher education. *Percentages followed by the same letter (a, b, c, d) do not differ at the 5% level.

Among respondents who considered the information received about each vaccine to be sufficient, 74.4% also reported the information about the diseases they prevent to be sufficient. On the other hand, among respondents who considered the information about vaccines to be insufficient, 45.7% also reported the information about the diseases they prevent to be insufficient. In Table 3, the answers are distributed according to the educational level of parents, and address doubts and attitudes towards the decision to vaccinate the child. Table 4 presents safety and risks perceptions.

**Table 3.** Concerns, doubts and attitudes towards the decision to vaccinate the child or not according to the educational level of parents with children up to 72 months of age.

Question	Responses (%)	Educational level*						Overall
		IES	CES	IHS	CHS	IHE	CHE	
Are they too many vaccines?	Yes	21.3^a^	20.2^a^	26.2^a^	19.3^a^	25.3^a^	21.7^a^	21.5
	No	78.7	79.8	73.8	80.7	74.8	78.3	78.5
I thought a lot about vaccinate	I didn’t think much	52.8	58.7	61.5	57.1	58.1	52.2	56.5
	I thought a lot and I agree	16.7^a^	8.7^ab^	8.4^ab^	6.5^b^	7.2^b^	8.8^ab^	8.2
	I didn’t think about	30.6^a^	32.6^a^	30.1^a^	36.4^a^	34.7^a^	38.9^a^	35.4
As to your certainty about vaccinating your child	# I was not sure and so I did not vaccinate	2.8	1.0	1.2	1.1	2.0	0.34	1.1
	# I was sure and I did not vaccinate	1.4	2.2	1.2	1.3	2.0	0.4	1.2
	# I was not sure and I postponed some vaccines	5.6	2.2	1.8	2.4	4.1	2.7	2.7
	I was not sure but I vaccinated	13.9	11.8	14.5	11.2	12.2	8.5	11.3
	I was sure and vaccinated	76.4^a^	82.8^ab^	81.3^ab^	84.1^ab^	79.6^ab^	88.1^b^	83.8
About your plans for vaccinating your child	Will receive all	90.4^a^	91.1^a^	96.3^a^	95.1^a^	92.9^a^	94.2^a^	94.3
	Will receive if I think they are necessary	8.2	6.7	3.7	4.1	5.1	5.8	5
	Will not receive all vaccines	1.4	2.2	0.0	0.9	2.0	0.0	0.8**

IES: incomplete elementary school; CES: complete elementary school; HIS: incomplete high school; CHS: complete high school; IHE: incomplete higher education; CHE: complete higher education; *percentages followed by the same letter (a, b) do not differ at the 5% level; #grouped for classification as hesitant; **88.9% reported the vaccine to be up to date.

**Table 4.** Source of information, safety, risks and mandatory vaccination of the child: perception of parents with children up to 72 months of age according to educational level.

Question	Responses (%)	Educational level*						Overall
		IES	CES	IHS	CHS	IHE	CHE	
Where do you look for information?	SUS	81.3^ab^	87.1^a^	80.8^ab^	74.9^b^	62.6^c^	37.0^d^	66.8
	Pediatrician	0.0	1.1	0.6	2.3	5.1	12.7	4.6
	Various sources	18.7^ab^	11.9^b^	18.6^ac^	22.7^ac^	32.3^ac^	50.3^d^	28.7
The immune system could be weakened by too many vaccines.	I agree	24.4	19.1	20	22.2	20.6	15.3	19.9
	I do not agree	35.1^a^	50.0^abc^	46.1^ab^	55.1^bc^	62.9^c^	76.3^d^	58.1
	I don’t know	40.5^a^	30.9^ab^	33.9^a^	22.8^b^	16.5^bc^	8.5^d^	22
The benefits of vaccines outweigh their risks.	I strongly agree	42.5^ab^	53.2^ab^	42.9^a^	45.9^a^	57.6^b^	70.7^c^	52.9
	I partially agree	15.1	21.3	22.0	24.5	30.3	24.6	23.8
	I do not agree	4.1	1.1	1.2	3.0	4.0	0.7	2.2
	I don’t know	38.4	24.5	33.9	26.6	8.1	4.1	21.1
How safe do you think vaccines are?	Very safe	67.1^a^	66.7^a^	62.5^a^	57.1^a^	62.7^a^	55.6^a^	59.3
	Safe	1.4	3.2	4.8	3.8	3.0	2.0	3.3
	Fairly unsafe	0.0	0.0	1.2	1.3	1.0	0.7	0.9
	Unsafe	31.5	30.1	31.6	37.7	33.3	41.7	36.5
Have you ever felt obligated to vaccinate your child?	Yes. I felt obligated (job. health service. school)	31.0	23.3	27.0	19.2	19.2	11.6	19.4
	I did not feel obligated	69.0a	76.7ab	73.0abc	80.8bc	80.8cd	88.4d	80.6

IES: incomplete elementary school; CES: complete elementary school; HIS: incomplete high school; CHS: complete high school; IHE: incomplete higher education; CHE: complete higher education; SUS: Unified Health System, the Brazilian public health system; *percentages followed by the same letter (a, b, c) do not differ at the 5% level.

Of the 163 respondents who wrote at least one question to the authors, the percentage of vaccine-hesitant individuals was significantly higher than that of non-hesitant individuals: 22.1% wanted more information about vaccines, and 7.9% asked why some vaccines were only available in private clinics and were not offered by the Unified Health System (SUS), which is the Brazilian public health system.

## DISCUSSION

This study permitted us to know the local reality and it will enable to assess changes in behavior at different times, such as in the post-pandemic future. In Brazil, the immunization program guarantees free access to vaccines for all children, which is not the case in other countries, and the comparison or extrapolation of the data must, therefore, consider this fact.

In the present study, 65% of the questionnaires were completed. Kennedy et al. reported the same percentage in a national survey conducted in the United States. Likewise, the authors did not evaluate the reasons why parents did not participate in the survey, which is considered a limitation of this type of study once the objective is to identify the different factors associated with vaccine hesitancy or refusal.^6^ It is necessary to continuously assess concerns and attitudes towards vaccines for effective communication with parents, since, as observed in this study, positive comments about the importance of vaccines are accompanied by doubts, concerns, hesitancy, and inconsistent responses. The significant differences between levels of education are part of the complex network of determinants in the decision whether or not to vaccinate; however, the concerns of each individual must be addressed in the planning of actions designed to maintain and increase vaccine acceptance.

In different societies, even with different levels of educational attainment, the decision to vaccinate children is shared among family members or is considered a task for the mother, who will make this decision based on the information she has and/or the information provided by the health service. Although sociocultural and economic issues may explain this conduct, measures encouraging parental participation are needed.^5^ Regardless of educational level, the mother is the person who takes the child to be vaccinated. The health service where the child receives the vaccine is considered the main source of information, in addition to the records on the vaccination card. Most parents, regardless of educational level, reported that their child is vaccinated at SUS and that they follow the vaccination instructions of the health services, which can be understood as confidence in the service.^4^ It is not possible to state whether the confidence in the service or the lack of efficient communication would be related to the fact that most respondents did not know how many vaccines their children had received and against which diseases the child would be protected. Regarding the communication between health services and parents, we observed that the information about vaccines received was not always considered to be sufficient. Thus, the best time for this educational intervention is controversial. According to Dubé et al. in Canada, providing this information in the maternity ward would have a limited range.^9^ Anxiety, tension and crying, which are common during vaccine administration, can also contribute to less efficient communication. This fact may explain in part that one-third of the respondents reported some adverse event; however, 56.2% of them could not identify which vaccine caused the event. Other times and types of communication are necessary and must address the reality of each community. The groups with higher levels of educational attainment that considered the information about vaccines received in the health services to be insufficient, usually seek different sources for clarification, are less hesitant, and expand vaccinations to private services. These groups also identified adverse events more frequently, probably because of better access to information. The behavior of this population suggests that individuals who have more financial resources and more information understand that their children should receive other vaccines not available in the public system. SUS is the guarantee of vaccines for 97.9% of the respondents. The answers to the questions revealed possible inconsistencies, or latent doubts, expressed during the interview. In the group with a lower level of educational attainment, 67.9% could not say whether vaccines weaken the immune system, but on the other hand also stated that vaccines are very safe, which appears to be inconsistent. In France, Peretti-Watel et al. observed that respondents with different socioeconomic conditions were facing the same dilemmas regarding vaccines and, despite accepting vaccination, doubts persisted. The authors quoted a respondent: “I think I did the right thing… I hope I am not wrong”.^5^ As for certainty about vaccination, respondents who hesitated or refused it, corresponded to 5.0% of the present sample. However, it is necessary to consider the complexity of the context about confidence, complacency and convenience, factors that influence vaccine hesitancy, including past experiences and future expectations. Thus, if we define vaccine acceptance with doubts as a form of hesitancy, 16.3% of the respondents were hesitant regarding vaccination in the past, and 5.7% regarding future vaccination, suggesting that the previous experience was positive and initial hesitancy was overcome. Among Polish medical students, Zarobkiewicz et al. observed that 98.7% intended to vaccinate their children.^9^ Dubé et al. noted the same intention among 77.5% of Canadian mothers of newborns. However, only 50.0% of mothers who expressed vaccine insecurity would follow recommendations regarding the vaccine doses that the child should receive in the future, which would result in a larger number of children with vaccine delay.^10,11^ These observations reinforce the need for communication, confidence, and positive experiences. In the present study, the differences in the parents’ educational level and access to information about vaccines may partially explain the different perceptions of the risk to which the child and the community are exposed if they do not receive the recommended vaccines.

In Brazil, the recommended vaccines are mandatory and free of charge. Thus, an informed consent, as recently suggested for COVID-19 vaccination, would therefore be of little use. However, mandatory vaccination and punishment, such as not allowing the child to attend a daycare center, pose a dilemma, which should go beyond the current limits of health ethics and human rights, to increase citizen engagement and a better approach for addressing vaccine hesitancy.^12^ In the present study, the set of responses suggests that most respondents trust vaccines and receive them through the public health system (SUS). This fact is probably more important than mandatory vaccination and most individuals, therefore, do not feel obligated to vaccinate their children, but do so. Perhaps information changes customs more than laws through honest dialogue. Vaccine hesitancy, that can result in refusal, is a silent process. Flexibility would always be possible in cases in which non-vaccination does not represent relevant public health risks, if we consider that there is no vaccine against refusal or hesitancy.^13,14^

Knowing the local reality is a strength that will permit to better guide actions aimed at reducing the different types and intensities of vaccine hesitancy or refusal. In a review of 38 studies, most conducted in high-income countries, Ames et al. observed that parents wanted more information than they were receiving, including a wider variety of reliable and easily accessible media.^15^

The confidence in the Brazilian National Immunization Program (PNI), built over decades, could have been shaken if the misinformation regarding the introduction of a new vaccine spread by various means, as is the case of COVID-19, was not immediately addressed, which requires preparation and action by healthcare managers. In Israel, Dror et al. observed that the acceptance rate of a future vaccine against COVID-19 was similar between doctors (78.0%) and the general population (75.0%), but was lower among nurses (61.0%). However, when asked about whether they would vaccinate their children against COVID-19, doctors and nurses had lower rates (60,0% and 55.0%) than the general population (70.0%).^16^ In Brazil, websites providing trustworthy information are available for the general population and for health professionals, including websites of the Ministry of Health, the WHO, the Pan American Health Organization (PAHO), the Brazilian Society of Pediatrics (SBP), and the Brazilian Society of Immunization (SBIm). The media engagement to provide and elucidate information is also important, and a proactive communication strategy is needed to address misinformation and anti-vaccine events.^17^ Vaccine hesitancy must also be seen within a social and political context since disinformation for religious purposes or as populist policy is a major enemy of vaccines.^18^ Even if small, declines in vaccine coverage can have social and economic repercussions.^19^ Vaccine-hesitant groups are organized on social media and post large amounts of anti-vaccine messages on popular websites that can reach various countries. According to Wilson and Wiysonge, the percentage of the population who believes vaccines are unsafe increases as social media use increases, ranging from up to 10% among those who never use social media to 30% among those who frequently use social media.^20^ In our study, the percentage of parents who considered vaccines to be fairly unsafe or unsafe (4.2%) may seem low for the conditions evaluated on that occasion. However, in the near future, we do not know what the behavior of the younger population will be, with longer social media usage, regarding whether or not to vaccinate their children, when this shall be done. Wilson and Wiysonge highlight the need for coordinated actions to remove anti-vaccine content from social media platforms, as well as actions against national or foreign sources that intentionally promote disinformation campaigns.^20^

Surveys are important to assess and understand local realities, in order to enable future comparisons regarding what parents think about vaccinating or not their children, but they only represent those who decide to participate. Once risk factors are identified, it is necessary to formulate responses so that at least this group continues vaccinating their children. However, knowing what those who do not participate think is a challenge.^6^ Such information will improve communication between parents and health services. Further studies will be necessary after the pandemic period to define new strategies that reduce vaccine hesitancy.

Overall, in this group, decisions of whether to vaccinate or not, attitudes, concerns, information, and hesitancy were positive in favor of vaccines. Doubts regarding the risks, benefits, and safety of vaccines, as well as unfamiliarity and misinformation, were observed even among respondents who do not feel obligated to vaccinate their children. The answers were heterogeneous among the different levels of educational attainment, sometimes showing statistical significance. There was also inconsistency between the answers of the same respondent, demonstrating that the approach to better communication must consider these diversities. Parents have vaccinated and intend to continue vaccinating their children, but information, misinformation and anti-vaccine movements can change this decision.
